# Comments on Co-laborer

**Published:** 1876-12

**Authors:** 


The above letter is an extract from an old and esteemed friend. His suggestions seem so forcible and well timed, and accord so well with the views of other medical brethren whom we have consulted on the subject, that we have, after mature reflection, determined to make the proposed change, and to lessen the size and price of THE RECORD.

We confess that our desire has been to enlarge rather than to diminish the size of our journal, but the exceeding pressure of the times, and the fact that intimations have come up to us that the size of THE RECORD is more expensive than suits a majority of our readers, and that something cheaper will answer the purpose, at least until times change and medical men are better paid, has decided us to adopt the suggestion and to issue a journal at $2 per annum. To do this will require some reduction in size, but will not diminish materially the amount of practical matter to be published. The labor of condensing and gleaning will be much increased, but the editors will not shrink from its performance, and will see to it that the pith and cream of all that is useful, both in the home and foreign journals, shall be given to our readers.

Brethren, we will spare no efforts to do our part, and to furnish you with an ample equivalent for the subscription money. Will you stand by us in the adoption of the cash system? If so, inform us at once, renewing your subscription upon our new basis, and on the reception of the January number, send up promptly $2 00 in lieu of $3 00, as heretofore.

